# Dynamic changes in Shiga toxin (Stx) 1 transducing phage throughout the evolution of O26:H11 Stx-producing *Escherichia coli*

**DOI:** 10.1038/s41598-023-32111-8

**Published:** 2023-03-27

**Authors:** Bungo Yano, Itsuki Taniguchi, Yasuhiro Gotoh, Tetsuya Hayashi, Keiji Nakamura

**Affiliations:** Department of Bacteriology, Graduate School of Medical Sciences, Kyushu University, 3-1-1 Maidashi, Higashi-ku, Fukuoka, 815-8582 Japan

**Keywords:** Bacteriophages, Bacterial genetics

## Abstract

Shiga toxin (Stx) is the key virulence factor of Stx-producing *Escherichia coli* (STEC). All known Stxs (Stx1 and Stx2) are encoded by bacteriophages (Stx phages). Although the genetic diversity of Stx phages has frequently been described, systematic analyses of Stx phages in a single STEC lineage are limited. In this study, focusing on the O26:H11 STEC sequence type 21 (ST21) lineage, where the *stx1a* gene is highly conserved, we analysed the Stx1a phages in 39 strains representative of the entire ST21 lineage and found a high level of variation in Stx1a phage genomes caused by various mechanisms, including replacement by a different Stx1a phage at the same or different locus. The evolutionary timescale of events changing Stx1a phages in ST21 was also determined. Furthermore, by using an Stx1 quantification system developed in this study, we found notable variations in the efficiency of Stx1 production upon prophage induction, which sharply contrasted with the conserved iron regulated Stx1 production. These variations were associated with the Stx1a phage alteration in some cases but not in other cases; thus, Stx1 production in this STEC lineage was determined by differences not only in Stx1 phages but also in host-encoded factors.

## Introduction

Shiga toxin (Stx)-producing *Escherichia coli* (STEC) are important foodborne pathogens that cause a range of diseases from diarrhoea to severe haemorrhagic enteritis and sometimes life-threatening complications such as haemolytic uraemic syndrome (HUS) and encephalopathy^1,2^. Stx is the key virulence factor responsible for the onset of these severe diseases. This toxin belongs to the AB-type toxin family and is classified into two antigenically distinct toxins, Stx1 and Stx2. Both types include several subtypes (Stx1a, Stx1c, and Stx1d; Stx2a–Stx2k)^3–6^, and STEC strains can produce a single Stx subtype or a combination of these Stx subtypes. Although Stx2 is more toxic to mice than Stx1^7^, the opposite result was reported in rabbits^8^.

The *stx* genes are encoded by temperate bacteriophages (Stx phages), which are integrated into host chromosomes by site-specific recombination to enter the lysogenic state. Similar to most other temperate phages, Stx phages are induced when the SOS response is provoked in host cells by DNA-damaging agents, such as treatment with mitomycin C (MMC)^9^. In this process called prophage induction (PI), the activated RecA protein stimulates autocleavage of the CI repressor, resulting in excision of the prophage genome by the action of integrase and excisionase and phage replication by the expression of a set of early genes, including the* n* antiterminator gene and the *o* and *p* replication genes. The transcription of early genes results in the expression of protein Q, which acts as an antiterminator for late gene expression^10^. As the *stx* genes are located just downstream of the terminator on which Q acts, Stx production is induced upon PI^11–14^. While Sx2 production is almost exclusively dependent on PI^15–17^, Stx1 expression is also controlled by an iron-regulated system, as there is an intrinsic promoter for the *stx1* gene (*p*_stx1_) and a ferric uptake regulator (Fur) binding sequence locates between the − 35 and − 10 regions of *p*_stx1_^18,19^. Therefore, *stx1* is expressed under low iron (LI) conditions. Growth in LI media stimulates Stx1 production but does not induce cell lysis due to PI; therefore, the toxin accumulates in cells^20^. While LI-dependent Stx1 induction has been observed in many *stx1*-positive strains^15,20,21^, PI-dependent Stx1 induction has only been described in a limited number of strains, such as the O26:H11 strain H19^15,20,21^.

STEC O26:H11 is one of the major non-O157:H7 STECs that has recently become the most predominant STEC in Europe^22^. There are two major sequence types (STs) in the O26:H11 lineage, ST21 and ST29 (based on Achtman’s scheme of multi-locus sequence typing^23^). While recently isolated ST29 strains, which are further divided into three clades (C1, C2, and C3)^17^, carry only *stx2a*, ST21 strains harbour *stx1a* alone or *stx1a* and *stx2a*^24^. We previously analysed whole genome sequences (WGSs) of a global O26:H11 strain set (n = 520) and found that ST21 is separated into two clades, C1 and C2; furthermore, we found that ST21C1 emerged from ST29C2 and ST21C2 emerged from ST21C1^25^. This study further confirmed the high conservation of *stx1a* in ST21 and the sporadic distribution of *stx2a* in both ST29 and ST21. Based on these findings, we speculated that while Stx2a phages were repeatedly acquired by multiple O26:H11 lineages (mostly in the last 60 years), the common ancestor of ST21 acquired an Stx1a phage that has been stably maintained in ST21 strains^25^. However, Stx phage genomes are sometimes highly variable even between strains belonging to the same serotype or lineage^26–28^. Therefore, in this study, we performed a systematic analysis of Stx1a phages in O26:H11 ST21 strains to determine how the Stx1a phage is conserved in this lineage. Our analysis unveiled an unexpectedly high level of genomic diversity of the Stx1a phages in O26:H11 ST21 and the complex evolutionary history and dynamics of Stx1a phages in this lineage. The relationship between the variation in Stx1 phage genomes (subtypes of Stx1a phages) and the PI- and LI-dependent levels of Stx1 production in host strains was also explored.

## Results and discussion

### O26:H11 ST21 strains selected for analysis

**Figure 1:**
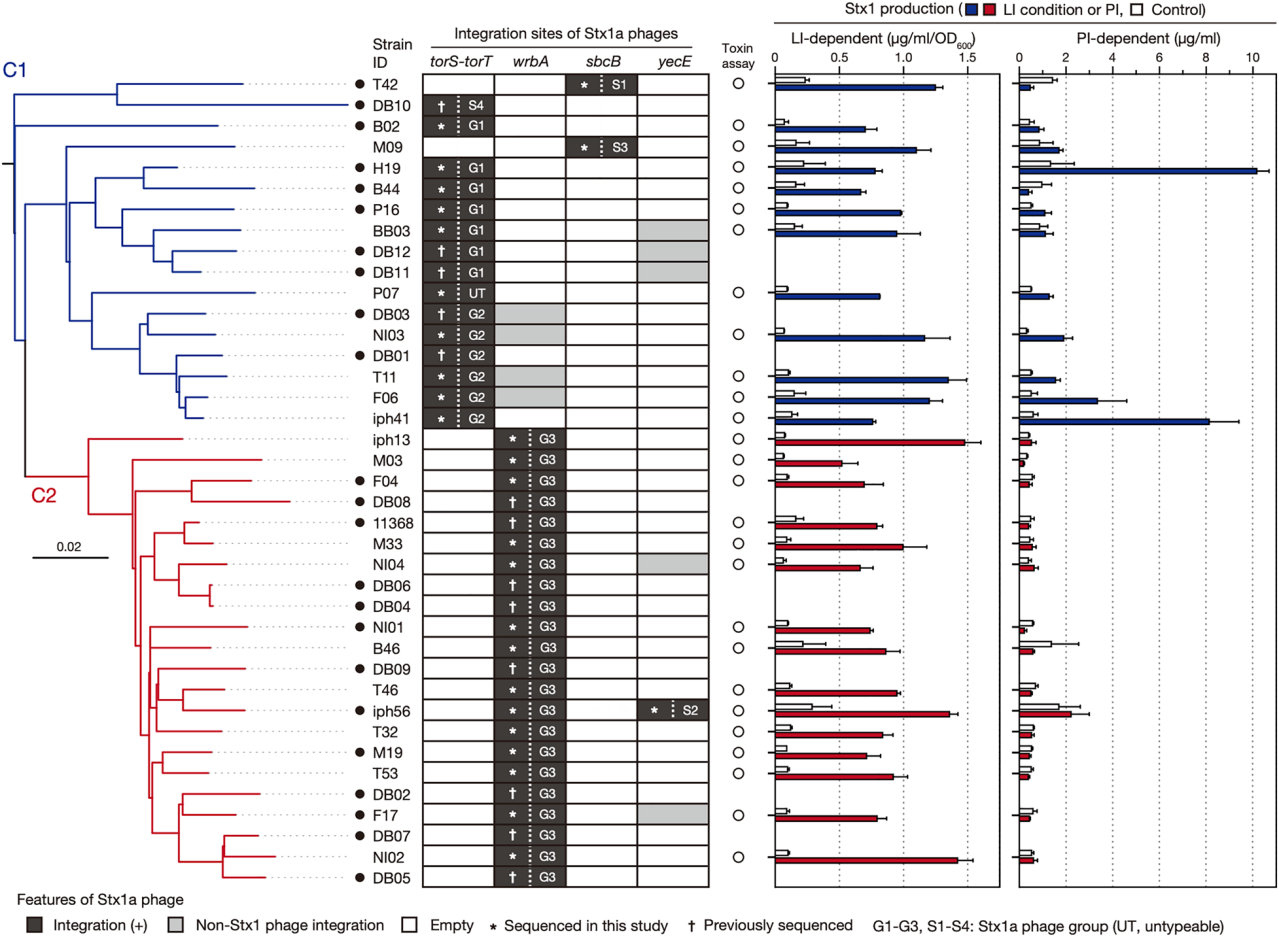
Variation in the integration site of Stx1a phages and Stx1 production between O26:H11 ST21 strains. A maximum likelihood (ML) tree of 39 O26:H11 ST21 strains is shown in the left panel. The tree was constructed based on the recombination-free SNPs (n = 3575) identified on the conserved chromosome backbone (4,340,670 bp) by RAxML using the GTR gamma substitution model. The route was determined by a similar phylogenetic analysis using an ST29C2 strain (090405) as an outlier. The bar indicates the mean number of nucleotide substitutions per site. Genome-closed strains are indicated by filled circles. Along with the tree, the chromosomal locations and types of Stx1a phages in each strain are shown. In the right panel, the levels of LI- and PI-dependent Stx1 production by each strain are shown as the mean values with standard deviations of biological triplicates or quintuplicates. Stx1 productions in all strains grown in the base medium were also measured as controls. Note that the Stx1 production in 12 strains, whose genome sequences were obtained from NCBI, were not determined.

To systematically analyse the Stx1a phages in O26:H11 ST21, we selected 27 ST21 strains from the 520 O26:H11 strains that we previously analysed^25^ and were available in our laboratory so that they covered the entire ST21 lineage as much as possible (Supplementary Fig. S1). The set of their genomes comprised one closed genome and 26 draft genomes, including both the ST21C1 and ST21C2 genomes. The full genome sequences of the Stx1a phage in 16 draft genomes were determined by a long PCR and Illumina sequencing after identifying their integration sites (see “Methods”). As those in the remaining 10 genomes were unable to determine by this strategy, their genomes were subjected to long-read sequencing by MinION (Oxford Nanopore Technologies; ONT) to generate closed genomes by hybrid assembly. In addition, we included 12 publicly available closed O26:H11 ST21 genomes in the analysis. Thus, the final set included the genomes of 39 strains (Fig. 1, Supplementary Dataset S1), of which 21 were isolated in Japan, and the remaining were isolated in the USA (n = 7), Belgium (n = 6), and other countries (n = 5) (Table 1). Most strains (n = 31) were human isolates, but four bovine and two environmental/food isolates were also included. The *stx* genotypes of these strains were *stx1a* alone (n = 20) or *stx1a* and *stx2a* (n = 18), while one strain carried two copies of *stx1a* and one copy of *stx2d* (strain iph56). We confirmed that the strain set was comprised of 17 ST21C1 and 22 ST21C2 strains by WGS-based phylogenetic analysis of the 39 strains (Fig. 1).

**Table 1:** The *stx* genotypes of O26:H11 strains analysed in this study.

Country	*stx1a*	*stx1a*/*stx2a*	*stx1a*(× 2)*/*stx2d*	Total
Japan	8	12	1	21
United States	4	3	0	7
Belgium	5	1	0	6
Other countries^†^	3	2	0	5
Total	20	18	1	39

*2 copies of *stx1a* genes.

^†^United Kingdom (n = 1), Germany (n = 1), Canada (n = 1), Argentina (n = 1), South Korea (n = 1).

### Variation in Stx1a phages

As the genome sequences of the Stx1a phages in 23 genome-closed strains were already available, we sequenced the Stx1a phage genomes of the remaining 16 strains to identify their integration sites and collect the full set of Stx1a phage genome sequences in the strain set (Fig. 1, Supplementary Dataset S1). As strain iph56 contained two Stx1a phages, genome sequences of a total of 40 Stx1a phages were obtained.

#### Variation in integration site

The Stx1a phages were 47.7–59.7 kb in length, with the exception of the phage in strain P07 (36.5 kb), which contained an insertion sequence (IS)-mediated ca. 18-kb deletion in the late region. All Stx1a phages were long-tailed phages with a set of late genes similar to that of phage lambda. Four loci (the *torS*–*torT* intergenic region, *wrbA*, *sbcB*, and *yecE*) were identified as the integration sites (Fig. 1). In the C1 strains (n = 17), 15 had Stx1a phages at *torS*-*torT,* and the remaining two had Stx1a phages at *sbcB*. In contrast, all C2 strains (n = 22) had Stx1a phages at *wrbA*, while an additional Stx1a phage of strain iph56 was present at *yecE*. Considering the phylogenetic relationship of the host strains, this finding suggested that changes in the Stx1a phage occurred in several C1 strains where the Stx1a phage at *torS*–*torT* changed to that at *sbcB* and in C2 strains where the Stx1a phage at *torS*–*torT* changed to that at *wrbA*.

#### Genome variation

**Figure 2:**
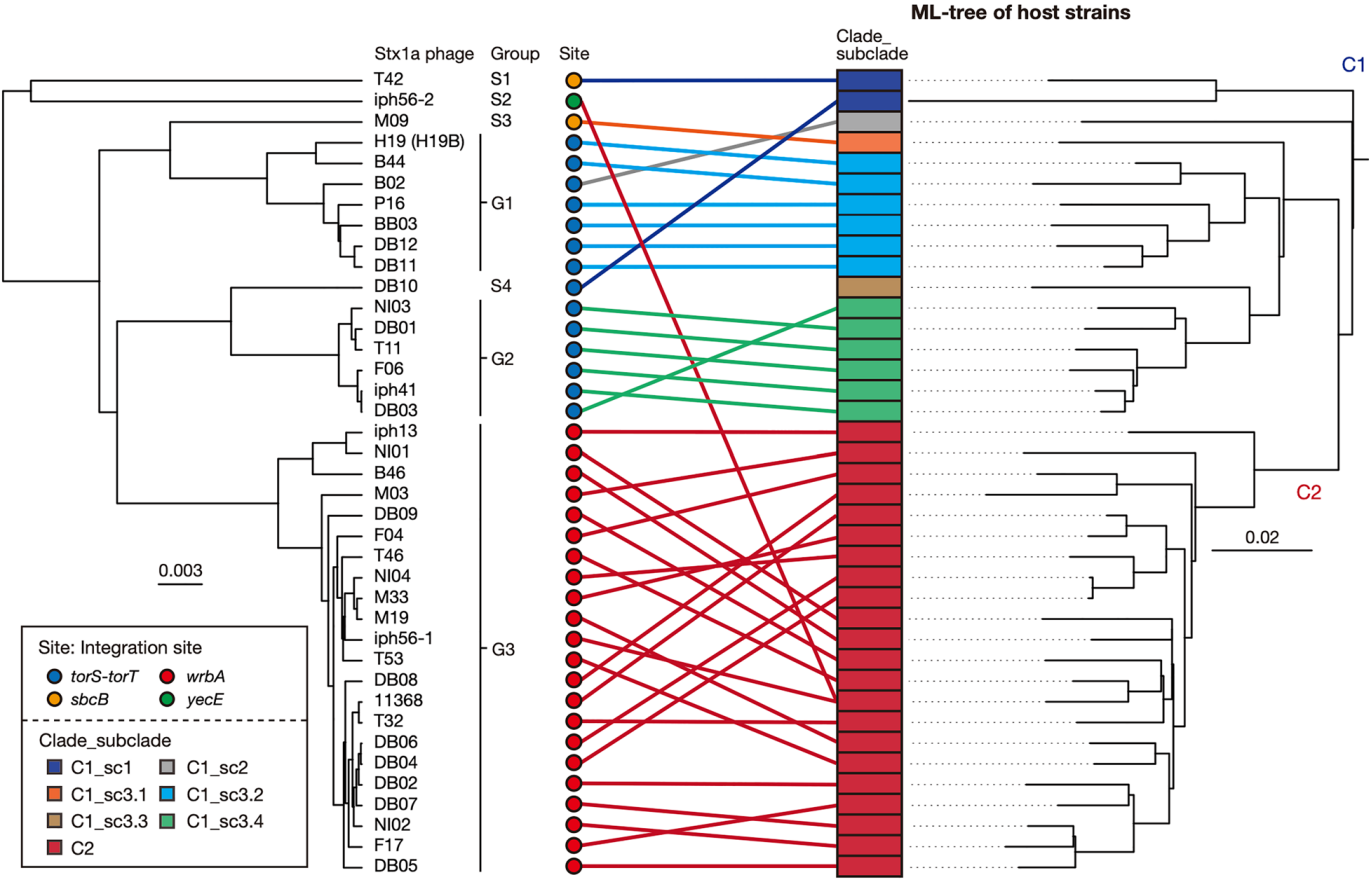
Sequence similarities between the Stx1a phage genomes found in the 38 O26:H11 strains. A UPGMA dendrogram based on the all-to-all Mash distance matrix of Stx1a phage genomes is shown in the left panel. The bar indicates the rate of sequence mutation estimated under a simple Poisson process of random site mutation (Mash distance)^29^. The right panel is the same ML tree of O26:H11 strains as shown in Fig. 1. Stx1a phages are connected to their host strains by lines coloured according to the clades of host strains. The degraded Stx1a phage genome in strain P07 (C1_sc3.3) was excluded from the analysis.

By complete sequence comparison of the 39 Stx1a phage genomes, excluding the abovementioned phage in strain P07, by the Mash program^29^ followed by clustering analysis based on the Mash distance matrix (Fig. 2, Supplementary Dataset S2), the Stx1a phages were classified into three groups (named G1, G2, and G3) and four singletons (S1–S4) with a maximum within-group Mash distance of 0.016. While all Stx1a phages at *wrbA* belonged to G3, those at *torS*-*torT* belonged to G1 or G2 (7 and 6 phages, respectively) and one singleton (S4). Three phages found at *yecE* or *sbcB* represented a singleton (S1–S3). Although the Stx1a phages within the same group indeed contained well-conserved genomes, certain degrees of variation were found in the late regions of several G1 and G3 phages (Supplementary Fig. S2). However, it appears that most of these variations were caused by chromosome inversion induced by multiple complicated recombinations between the Stx1a phage and other lambda-like prophages in each chromosome. Sequence comparison of seven Stx1a phages, representing the three groups and four singletons, showed clear differences in sequence between these phage genomes (Supplementary Fig. S3).

These sequence variations, together with the variation in the integration site, indicate that changes in the Stx1a phage repeatedly occurred throughout the evolution of ST21.

### Evolutionary history of the Stx1a phage in ST21

**Figure 3:**
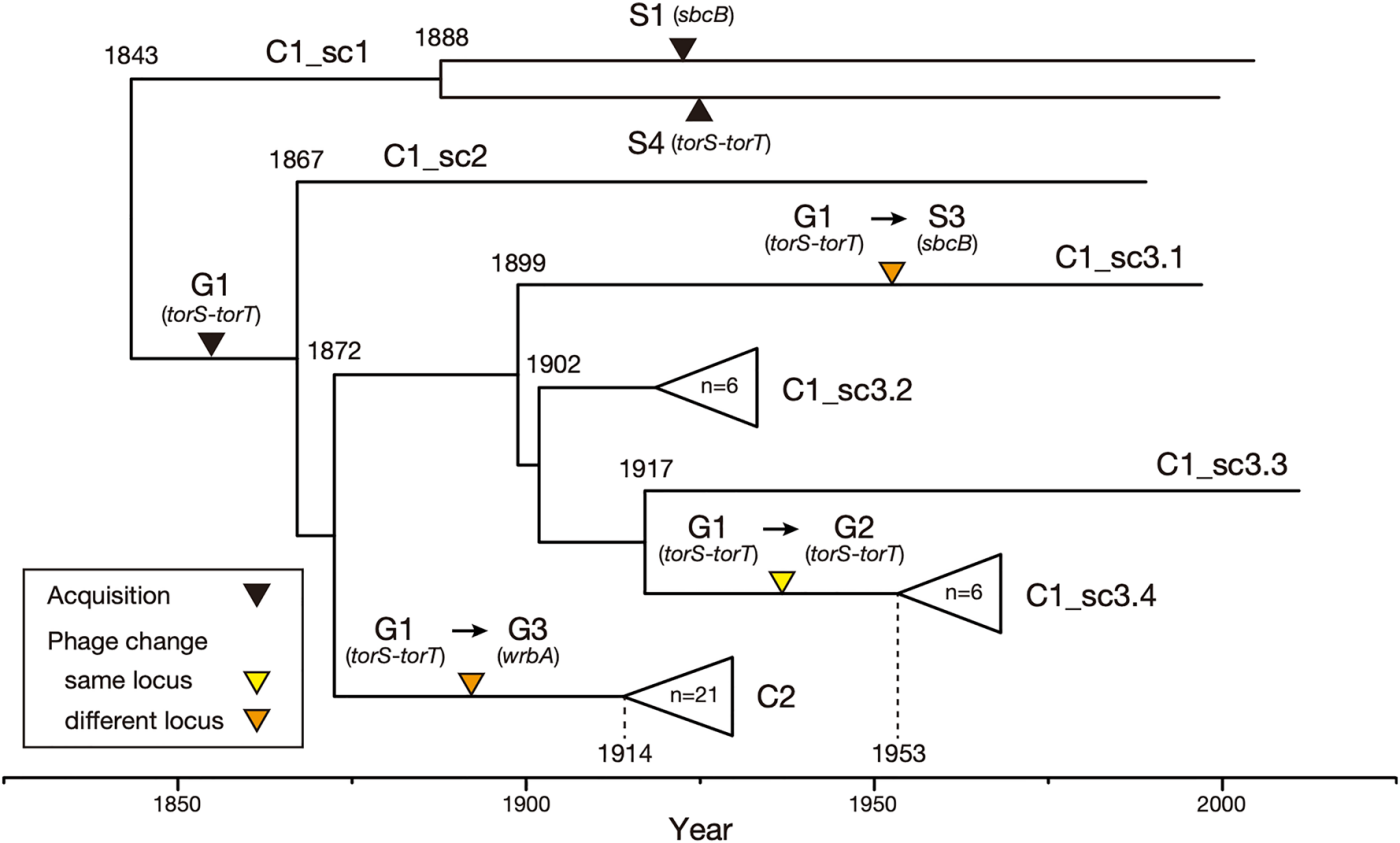
Timeline of the acquisitions and changes of Stx1a phages throughout the evolution of ST21. The estimated timing of acquisitions and changes to Stx1a phages are indicated by coloured triangles in a maximum clade credibility tree of ST21 strains. Tips, branches and internal nodes in C1_sc3.2, C1_sc3.4, and C2 are brought together under open triangles. A C2 strain (DB02) lacking temporal information was not included in this tree. The complete topology of the tree is shown in Supplementary Figure S4.

To understand the evolutionary history of the changes in the Stx1a phages in ST21, we compared the Mash distance-based clustering patterns of Stx1a phages and the phylogenetic relationships of their host strains (Fig. 2). For this analysis, as multiple changes in Stx1a phage occurred in C1, this clade was divided into three subclades sc1, sc2, and sc3, with further subdivision of sc3 into four groups sc3.1 to sc3.4, as shown in the right panel in Fig. 2. A temporal analysis of the ST21 strains by Bayesian coalescent analysis^30^ was performed to estimate the time to the most recent common ancestor (TMRCA) of each ST21 clade/subclade (Supplementary Fig. S4). The phylogenetic relationship of the ST21 strains indicated that C1_sc1 was the first separation in the ST21 lineage, followed by C1_sc2, and finally C1_sc3 and C2 emerged from C1_sc2 (Fig. 2). As summarized in Fig. 3, during this process, strains T42 and DB10 in C1_sc1 independently acquired S1 Stx1a phages at *sbcB* and S4 Stx1a phage at *torS*-*torT*, respectively. The common ancestor of C1_sc2 and C1_sc3 acquired a G1 phage at *torS*-*torT* between 1843 and 1867 (Fig. 3) because G1 phages at *torS*-*torT* were found in C1_sc2 which is a single-member subclade containing strain B2 and all C1_sc3.2 strains (Fig. 2). The presence of the S3 phage at *sbcB* in C1_sc3.1 which is also a single-member subclade containing strain M09 indicated the deletion of the G1 phage from *torS*-*torT* and the acquisition of the S3 phage at *sbcB* in this subclade. The presence of the G2 phage at *torS*-*torT* in all sc3.4 strains (Fig. 2) indicated that the replacement of G1 phage by G2 phage occurred at the *torS*-*torT* locus in C1_sc3.4 between 1902 and 1953 (Fig. 3). In C2, which formed a sister branch of C1_sc3, all strains had the G3 phage at *wrbA*, indicating that the deletion of the G1 phage and the acquisition of G3 phage occurred at *wrbA* between 1872 and 1914 (Fig. 3) upon the emergence of ST21C2, and the G3 phage has been stably maintained in this clade. The S2 phage at *yecE*, the second Stx1a phage of strain iph56, was further acquired by this C2 strain very recently, later than 1973 (not shown in Fig. 3, see Supplementary Fig. S4). Thus, most acquisition and changes in Stx1a phages in the ST21 lineage occurred between 1843 and 1953 (Fig. 3), in contrast to the fact that most *stx2* acquisition events in O26:H11 occurred in the last 60 years^25^.

The intra-lineage genetic diversity of Stx phages has been described for the O157:H7, O26:H11, O145:H28, and O121:H19 STEC lineages^26–28,31–33^. However, to the best of our knowledge, systematic and large-scale analyses of the genomic diversity of Stx phages have been performed only for the Stx2a phages in STEC O121:H19^33^ and clade 8 of STEC O157:H7^28^. In O121:H19, an Stx2a phage was acquired at *argW* by the common ancestor of the currently circulating major lineage (named L1) 80 years ago or earlier, and this phage has been very stably maintained in this lineage. In O157:H7 clade 8 strains, most of which carry *stx2a* plus *stx2c* or *stx2a* only, while the Stx2c phage is very stably maintained at *sbcB*, notable variations were observed in Stx2a phages. However, these variations were caused by the replacement of parts of the early regions of the Stx2a phages, all of which are located at *argW*. Therefore, the changes in the Stx1a phages in O26:H11 ST21 are more complicated and dynamic.

### Stx1 production levels in ST21 strains

While the Stx1a phage genomes in ST21 strains have highly diverged, the Fur-binding sequence was completely conserved in the *p*_stx1_ promoter of the Stx1a phages analysed in this study. To examine how the variation in the Stx1a phage genome affects the Stx1 production of host strains, we quantified the Stx1 production in 27 strains (12 C1 and 15 C2 strains) available in our laboratory after MMC treatment or cultivation in the presence of an iron chelator (2,2’-dipyridyl [2DPy]), each representing PI- and LI-dependent Stx1 induction, respectively. In addition, Stx1 production under noninducing conditions was also quantified (referred to as controls in Fig. 1) to evaluate the induction efficiency. For Stx1 quantification, we developed a homogeneous time-resolved fluorescence resonance energy transfer (HTRF) assay-based Stx1 quantification system similar to that we recently developed for Stx2^34^. To optimize the system, we selected the best monoclonal antibody (mAb) pair from a total of nine combinations (see Supplementary Fig. S5a for more details) and determined suitable antibody concentrations (Supplementary Fig. S5b) and incubation times (Supplementary Fig. S5c). The optimal 2DPy concentration (0.2 mM) and sampling time (4 h) were also determined by analyses using strains H19 and 11368, which represent ST21C1 and ST21C2, respectively (Supplementary Fig. S6).

**Figure 4:**
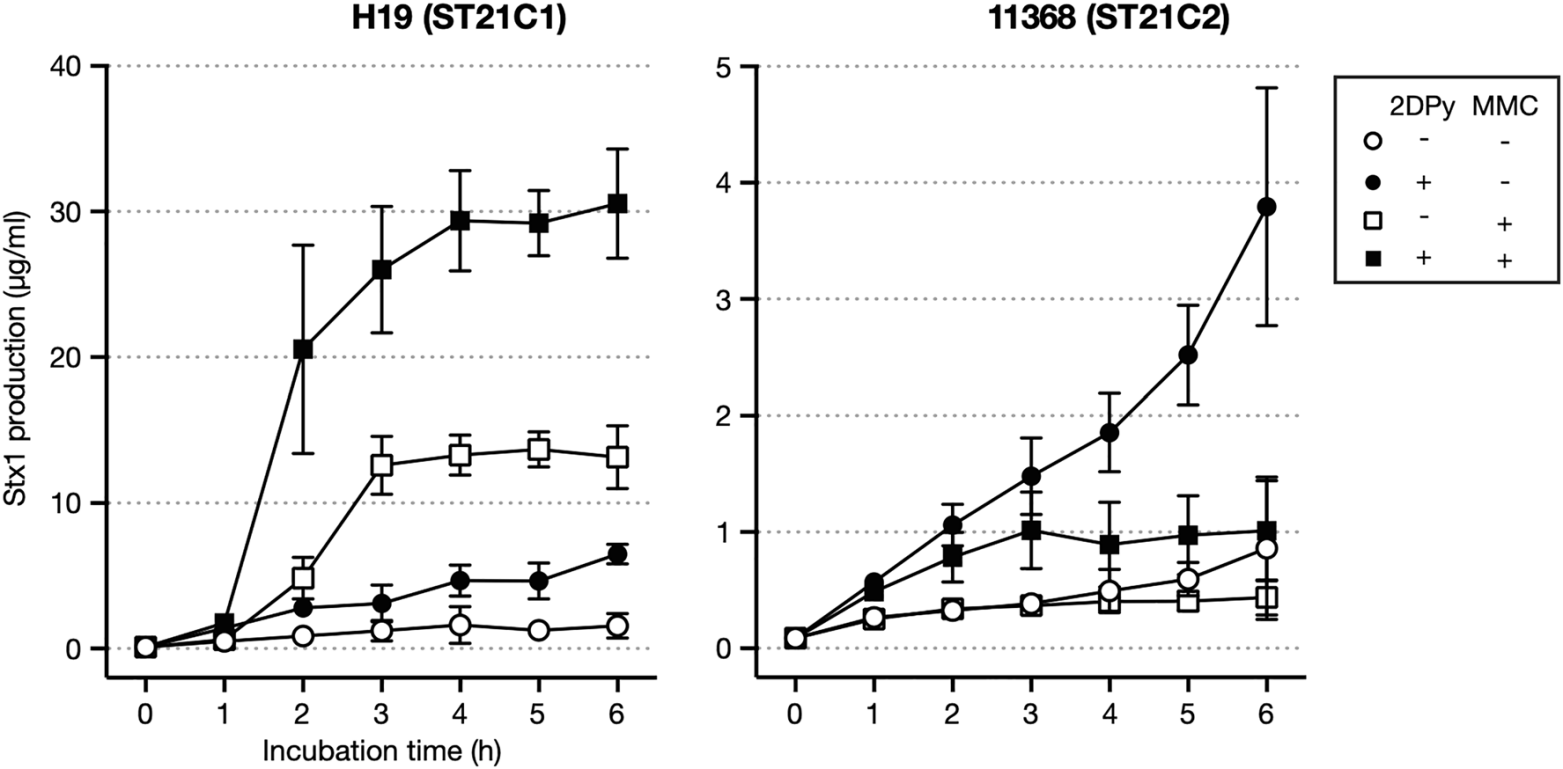
Stx1 production by strains H19 and 11368 in the presence of 2DPy, MMC or both. The Stx1 production levels of strains H19 (ST21C1) and 11368 (ST21C2) were determined. After adding each reagent to cultures grown to mid-log phase (T = 0 h), an aliquot of culture was collected every hour for 6 h to prepare cell lysates and their supernatants. Stx1 concentrations are shown as the mean values with standard deviations of biological triplicates.

As shown in Fig. 1 and Supplementary Dataset S1, Stx1 production in ST21 strains under LI conditions was 0.5–1.5 µg/ml/OD_600,_ with induction efficiencies ranging from 3.5× to 20×. Thus, although the LI-dependent Stx1 induction efficiency was variable, a similar level of Stx1 production was produced under the LI condition in all ST21 strains, which was consistent with the conservation of the Fur-binding sequence. In contrast, Stx1 production after MMC treatment was variable in the C1 strains (0.4–10 µg/ml) and low in all C2 strains (0.2–0.6 µg/ml), with the exception of strain iph56 (2.2 µg/ml), which carried an additional Stx1a phage. The PI-dependent Stx1 induction efficiency was between 2× and 14× in the C1 strains, except for three strains (T42, B44 and BB03; 0.3×, 0.4× and 1.3×, respectively), while that in the C2 strains was between 0.4× and 1.6×. This result indicated that Stx1 production is induced by PI in most C1 strains, but not in C2 strains. The analysis of Stx1 production by MMC treatment under LI conditions using strains H19 and 11368 suggested that Stx1 production is further enhanced under LI conditions in ST21C1 strains, but not in ST21C2 strains (Fig. 4).

Further comparison of PI-dependent Stx1 production between the C1 strains carrying the same Stx1a phage group, namely G1 phage-carrying strains H19, B44, BB03 and G2 phage-carrying strains T11, F06, iph41, revealed that the induction efficiency was also variable within each group, which was 0.4×–7.6× in the G1 group and 3.1×–14× in the G2 group. This result indicated that there are significant differences in the efficiency of PI-dependent Stx1 induction even between the strains carrying similar Stx1 phages.

### Comparison of the early regions of Stx1a phage genomes

**Figure 5:**
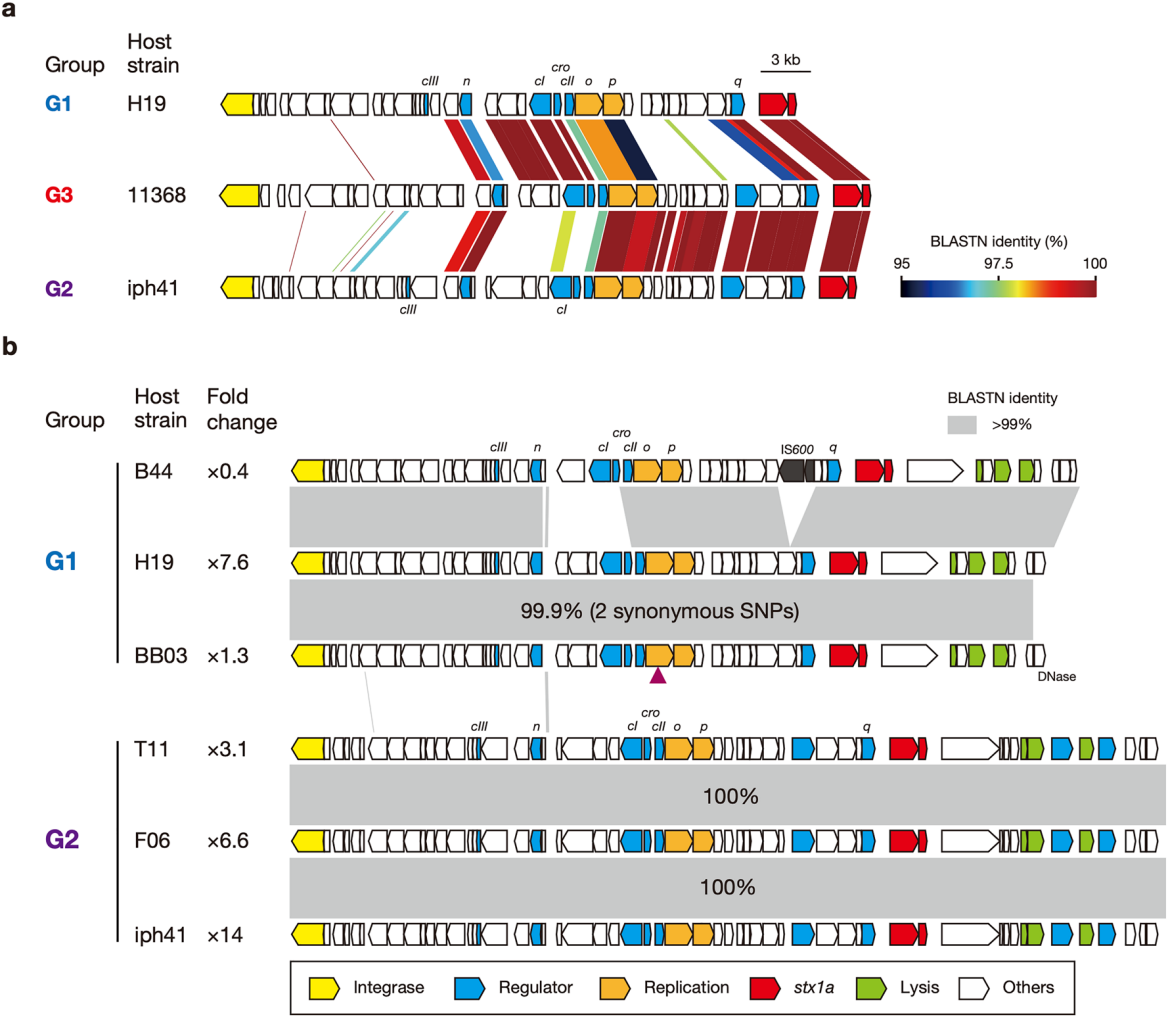
Comparison of the early regions of Stx1a phages. (**a**) Genomic organizations of the early regions of three Stx1a phages, representing the G1, G2 and G3 groups. The levels of nucleotide sequence identities between coding sequences (CDSs) are indicated by a heatmap. (**b**) Genomic organizations of the early regions of the G1 and G2 phages. Homologous regions showing > 99% nucleotide sequence identity are depicted by shading. Fold change indicates the efficiency of PI-dependent Stx1 induction (the Stx1 concentrations relative to the control). The presence of two synonymous SNPs found in the Stx1a phage of strain BB03 is indicated by a purple triangle.

PI-dependent Stx1 production can also be achieved by the expression of early genes^12,13^. Therefore, to examine the relationships between the variation in the Stx1 phage genomes and the difference in the PI-dependent Stx1 production between ST21 strains, we directly compared the genomic sequences of the early regions of three groups of Stx1 phages (Fig. 5a). This analysis revealed that the gene cluster including five genes (*cro*, *cI*, and three genes upstream *cI*) and the *q* gene were identical in sequence between G1 and G3, while the *n* gene and the gene cluster from *o* to *q* were identical or nearly identical between G2 and G3. Thus, the early region (from *n* to *q*) of the G3 phage was a chimaera generated by recombination between G1- and G2-like phages. This difference in genomic structure between the G3 phage and the G1 and G2 phages (recombination-induced structural changes and/or variations in sequence) may be linked to the lack of PI-dependent Stx1 induction in the ST21C2 strains.

As mentioned above, while PI-dependent Stx1 induction was observed in most strains carrying G1 or G2 phages, notable variations in induction efficiency were detected for strains carrying each group of Stx1 phages (Fig. 1). Therefore, we further analysed the intragroup variation in the early regions in each group (the G1 phages in strains H19, B44, and BB03 and the G2 phages in strains T11, F06, and iph41; Fig. 5b). Among the G1 phages, compared to the Stx1 phage in strain H19 (PI-dependent Stx1 induction; 7.6×), a replacement of a small region including the *cI* and *cro* genes and an IS*600* insertion in the upstream region of the *q* gene occurred in strain B44, which may be responsible for the lack of PI-dependent Stx1 production in this strain (0.4×). However, the Stx1 phage of strain BB03, which also showed low or almost no PI-dependent Stx1 induction (1.3×), contained an almost identical early region to that in strain H19; there were only two synonymous SNPs in the *o* gene. Although the early regions of the three G2 phages were completely identical (Fig. 4b), a certain level of variation in the PI-dependent Stx1 induction efficiency was also observed in their host strains (from 3.1× to 14×). These results indicated that the variation in Stx1a phage genomes does not explain the difference in PI-dependent Stx1 induction efficiency observed in the strains belonging to ST21C1.

## Conclusion

Our systematic analysis of Stx1a phages in the O26:H11 ST21 lineage revealed the dynamic changes in Stx1a phages throughout the evolution of this STEC lineage, which included not only the replacement of parts of the phage genome but also the loss of the existing Stx1a phage, and gain of a different Stx1a phage at the same or different chromosome loci. Quantification of the Stx1 induction levels of host strains using the HTRF-based assay developed in this study further revealed that while ST21 strains exhibited a similar level of LI-dependent Stx1 production, ST21C1 strains showed variable levels of PI-dependent Stx1 production, and ST21C2 strains showed no PI-dependent Stx1 production. The early regions of Stx1 phages in ST21C2 strains (G3 phages) differed from those in ST21C1 strains, which may be related to the lack of PI-dependent Stx1 induction in this subclade. However, notable variations in the PI-dependent Stx1 induction efficiency were also observed in C1 strains carrying very similar or identical Stx1a phages (G1 or G2 phages). Therefore, to understand the mechanisms underlying these variations between ST21 strains, not only the difference in Stx1a phages but also that in host chromosome- or other prophage-encoded factors affecting the induction of Stx1 phage need to be analysed.

## Methods

### Bacterial strains

The O26:H11 strain set included 27 strains available in our laboratory that were genome sequenced in our previous study^25,35^ and 12 strains with publicly available closed genome sequences. To obtain the genome sequence of the 12 strains, we downloaded all closed genomes of *E. coli* from the NCBI database (last accessed: April 15, 2022) and selected those of O26:H11 ST21 using ECTyper^36^ and MLST2.0 (https://cge.food.dtu.dk/services/MLST/). The final set was comprised of 38 ST21 strains and one strain belonging to ST7916, a single locus variant of ST21, as listed in Supplementary Dataset S1.

### Hybrid assembly for closing the genome

To close the draft genomes of 10 strains, their genomic DNA was purified using Genomic-tip 100/G (Qiagen) and sequenced using MinION with R9.4.1 flow cells (ONT) for 16–48 h. Read data in FASTQ format were generated using Guppy v4.0.15, v5.0.17, or v6.0.6 (ONT), trimmed by porechop (v0.2.2)^37^ with default parameters and filtered by NanoFilt (v2.7.1)^38^ with a threshold of > 1 kb at a quality score of > 8 (strain iph56), > 12 (strain F04), or > 10 (the other strains). The Illumina reads previously obtained for these strains^25^ were trimmed using Platanus_trim v1.0.7 (http://platanus.bio.titech.ac.jp/pltanus_trim). The filtered long reads were assembled along with the trimmed Illumina reads of each strain using the microPIPE pipeline^39^. As the chromosomes of strains F04, B02 and P16 were not circularized and that of strain T42 was split into two contigs, these chromosomes were gap-closed using Circlator v1.5.5^40^ with the long reads of each strain, followed by sequence correction by NextPolish v1.4.0^41^ using Illumina reads. As tandemly duplicated genomes were generated for the small plasmids (ca. 7 kb) in strains B44, F04, iph56, and M19, these sequences were manually corrected.

### Analyses of integration sites and sequencing of Stx1a phages

To determine the full genome sequences of Stx1a phages in the 16 draft genomes, we first examined the integration of the Stx1a phage into the five known integration loci for Stx1 phages (*torS*-*torT*, *wrbA*, *yehV*, *sbcB*, and *yecE*)^42,43^ by BLASTN and long PCR, a similar strategy to that we previously employed for the analysis of Stx phages in STEC O145:H28 (schematically shown in Supplementary Fig. S7a)^44^. Then, the entire genome sequences of each Stx1a phage were determined by sequencing long PCR products with the strategy shown in Supplementary Figure S7b.

### Phylogenetic analysis

Phylogenetic analysis of the ST21 strains (n = 39) was performed based on the SNPs identified on the prophage/integrative element/IS-free and recombination-free chromosome backbone that were conserved across all genomes; this analysis was performed by Gubbins^45^ and MUMmer^46^ using strain 11368 as a reference. A maximum likelihood (ML) tree was constructed with RAxML^47^ as previously described^25^ and displayed using FigTree v1.4.4 (http://tree.bio.ed.ac.uk/software/figtree/).

### Temporal analysis

By excluding one strain (DB02) whose temporal information was not available, 38 strains were selected, and an ML tree was constructed using 3311 recombination-free SNP sites in their conserved chromosome backbone sequence (4,353,874 bp in length) by the same method described above. The temporal signal in the tree was confirmed using TempEst^48^ by assessing the linear relationship between the root-to-tip distance and the year of isolation. Four combinations of different clock types (strict clock and uncorrelated relaxed clock) and population models (constant and exponential growth) in the GTR substitution model were compared by assessing the Bayes factor, and the strict clock and constant population size model was selected as the best-fit model. Using this model, a temporal analysis was performed using BEAST v1.8.4^30^ as previously described^25^. The result was summarized as a maximum clade credibility tree using TreeAnnotator in BEAST and visualized with FigTree v1.4.4.

### Comparison of Stx1a phage genome sequences

Complete phage genome comparison was performed using Mash v2.0^29^ with default parameters to generate pairwise Mash distance matrices. Based on the matrix, Stx1 phages were clustered by the UPGMA algorithm, and dendrograms were generated with the hclust command in R^49^. Stx1 phage genomes were annotated by DFAST^50^. GenomeMatcher v3.0.4^51^ was used for the genetic structure and sequence comparison of Stx1a phages.

### Development and optimization of the HTRF assay for quantification of Stx1

To construct an Stx1 quantification system, we employed an HTRF assay (Cisbio/PerkinElmer). To develop the HTRF assay, we used a commercially available lyophilized Stx1 preparation (Verotoxin 1; Denka Seiken) as the standard and the following three mAbs specific to Stx1: 9L400 (anti-Stx1, US Biological), VT004 (anti-Stx1 B-subunit; Nacalai Tesque), and C137686 (anti-Stx1 A-subunit; Lifespan Biosciences). Abs used as donors were labelled with europium (Eu) cryptate or terbium (Tb) cryptate, while acceptor Abs were labelled with d2 (an organic dye with the same photophysical properties as XL655). Delta F (DF) values (%) of each reaction were calculated for the interassay comparison following the procedure described on the CisBio website (https://www.cisbio.jp/content/signal-treatment-and-analysis/). Assay conditions were optimized by determining the best combination of fluorescence-labelled mAbs, the best concentrations of the mAbs, and the best incubation time as follows (Supplementary Fig. S5): 0.5 µg/ml Tb-labelled 9L400 mAb, 0.25 µg/ml d2-labelled VT004 mAb, and 18 h incubation. The working range of the HTRF assay was between 0.8 and 100 ng/ml (Supplementary Fig. S5c). After incubation for 18 h at room temperature, emissions were measured at 665 nm and 620 nm by a microplate reader (Infinite 200 PRO [TECAN]). Based on the 665 nm and 620 nm counts, Stx1 concentrations in each sample were calculated with a standard curve using Prism 9 software (GraphPad Software) as previously described^34^. Under these assay conditions, Stx1 concentrations were determined by the same procedure as previously described^34^.

### Quantification of Stx1 production by ST21 strains

Overnight cultures of each strain were inoculated into 2 ml of modified syncase broth (MS broth; 35 mM Na_2_HPO_4_, 29 mM K_2_HPO_4_, 22 mM NH_4_Cl, 0.63 mM Na_2_SO_4_, 0.21 mM MgCl_2_·6H_2_O, 20 µM MnCl_2_·6H_2_O, 18 µM FeCl_3_·6H_2_O, 20 g/l Bacto™ Casamino Acids [Thermo Fisher], 5 g/l Sucrose [Sigma‒Aldrich], 2 g/l d-(+)-Glucose [Nacalai Tesque], 40 mg/l tryptophan [Wako])^52^ at an OD_600_ of 0.1 and grown to mid-log phase at 37 °C with shaking. Then, 2DPy (Sigma) or MMC (Wako) was added to the cultures at a final concentration of 0.2 mM or 0.5 µg/ml, respectively. The 2DPy concentration and sampling time of bacterial cultures were optimized based on the results of exploratory analyses using strains H19 and 11368 (see Supplementary Fig. S6 for details). After 2DPy or MMC addition, 300 µl of culture was collected and subjected to sonication using Bioruptor (Cosmo Bio) for 5 min (four sonication cycles for 75 s with intervals for 30 s) in ice water. Then, the soluble fractions of each lysate were collected via centrifugation at 7700×*g* for 10 min at 4 °C. Samples were also prepared from the cultures grown in MS broth without 2DPy and MMC as controls. The Stx1 concentration in each sample was determined by the HTRF assay described above. When the coefficient of variation (standard deviation per mean value) obtained by the biologically triplicate experiments was > 0.4, two more experiments were performed.

To determine the level of Stx1 production induced by PI under LI conditions, we analysed two strains, representing ST21C1 and ST21C2 (strains H19 and 11368). These strains were grown in MS broth to mid-log phage at 37 °C with shaking, and 2DPy and/or MMC were added to the cultures. Then, an aliquot of culture was collected every hour for 6 h, and cell lysates and their supernatants were prepared as described above.

### Ethical approval

There are no ethical considerations applicable to the work presented.

## Supplementary Information

Supplementary Information 1.

Supplementary Information 2.

Supplementary Information 3.

## Data Availability

The data generated or used in this study are included in the main text or supplementary information of this article. The closed genome sequences of the 10 O26:H11 strains that were obtained in this study have been deposited in DDBJ/EMBL/GenBank under BioProject accession numbers starting from PRJDB14896 (https://www.ncbi.nlm.nih.gov/bioproject; see Supplementary Dataset S1 for accession numbers of each strain). All Stx1a phage genome sequences determined in this study have been deposited in the DDBJ/EMBL/GenBank databases under the accession numbers listed in Supplementary Dataset S1.
