# Supplementary material for: Dynamic changes in Shiga toxin (Stx) 1 transducing phage throughout the evolution of O26:H11 Stx-producing Escherichia coli
Source: Sci Rep. 2023 Mar 27;13:4935. doi: 10.1038/s41598-023-32111-8 (PMC10042803; doi:10.1038/s41598-023-32111-8)
Supplement: Supplementary file 1 — Supplementary Information 1. [file 41598_2023_32111_MOESM1_ESM.pdf]

Supplementary Information

Dynamic changes in Shiga toxin (Stx) 1 transducing phage throughout the evolution of O26:H11 Stx-producing *Escherichia coli*

Bungo Yano, Itsuki Taniguchi, Yasuhiro Gotoh, Tetsuya Hayashi, Keiji Nakamura

Department of Bacteriology, Graduate School of Medical Sciences, Kyushu University, 3-1-1 Maidashi, Higashi-ku, Fukuoka 815-8582, Japan

E-mail: nakamura.keiji.046@m.kyushu-u.ac.jp

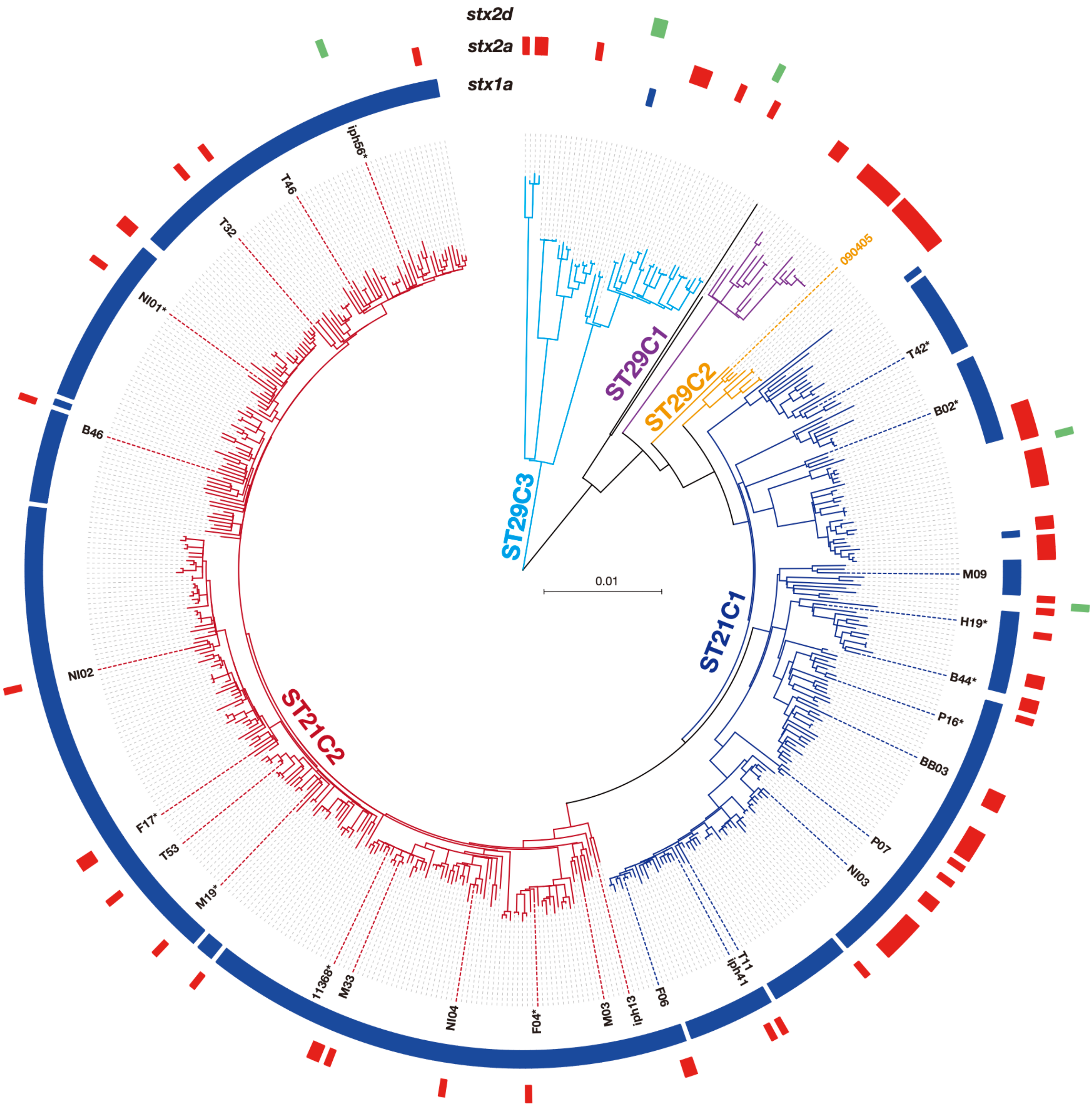

Figure S1. Positions of 27 laboratory strains in the O26:H11 phylogeny.

In the maximum likelihood (ML) tree of 520 O26:H11 strains, which we previously constructed [Ogura *et al. Microb Genom*, 3(11), e000141 (2017)], the 27 strains used in this study are indicated. The distributions of *stx1a*, *stx2a*, and *stx2d* are also shown. The 11 genome-closed ST21 strains (5 C1 and 6 C2 strains) are indicated by asterisks. The outlier used in the ML-tree in Fig. 1 is indicated in orange. The 12 closed genomes obtained from the public database were not included in the tree.

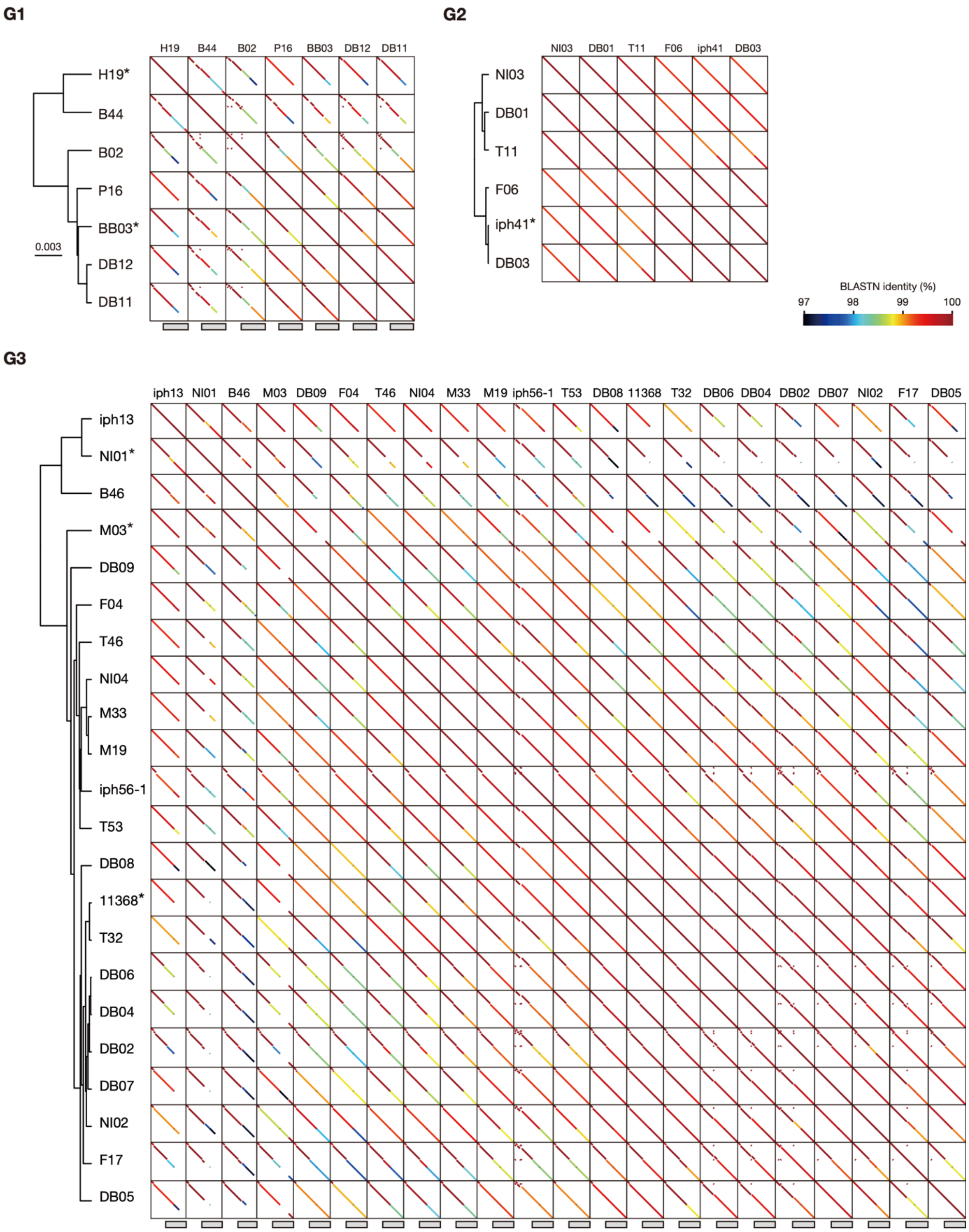

Figure S2. Within-group sequence conservation of Stx1a phage genomes.

Along with the dendrogram of each phage group based on the Mash distance matrix (parts of the dendrogram shown in Fig. 2), dot plot presentations of the sequence similarity of Stx1a phage genomes are shown with a heatmap (>97% sequence identity). The phages compared in Supplementary Fig. S3 are indicated by asterisks. The late regions of the G1 and G3 phages are indicated by grey rectangles at the bottom of each dot plot matrix.

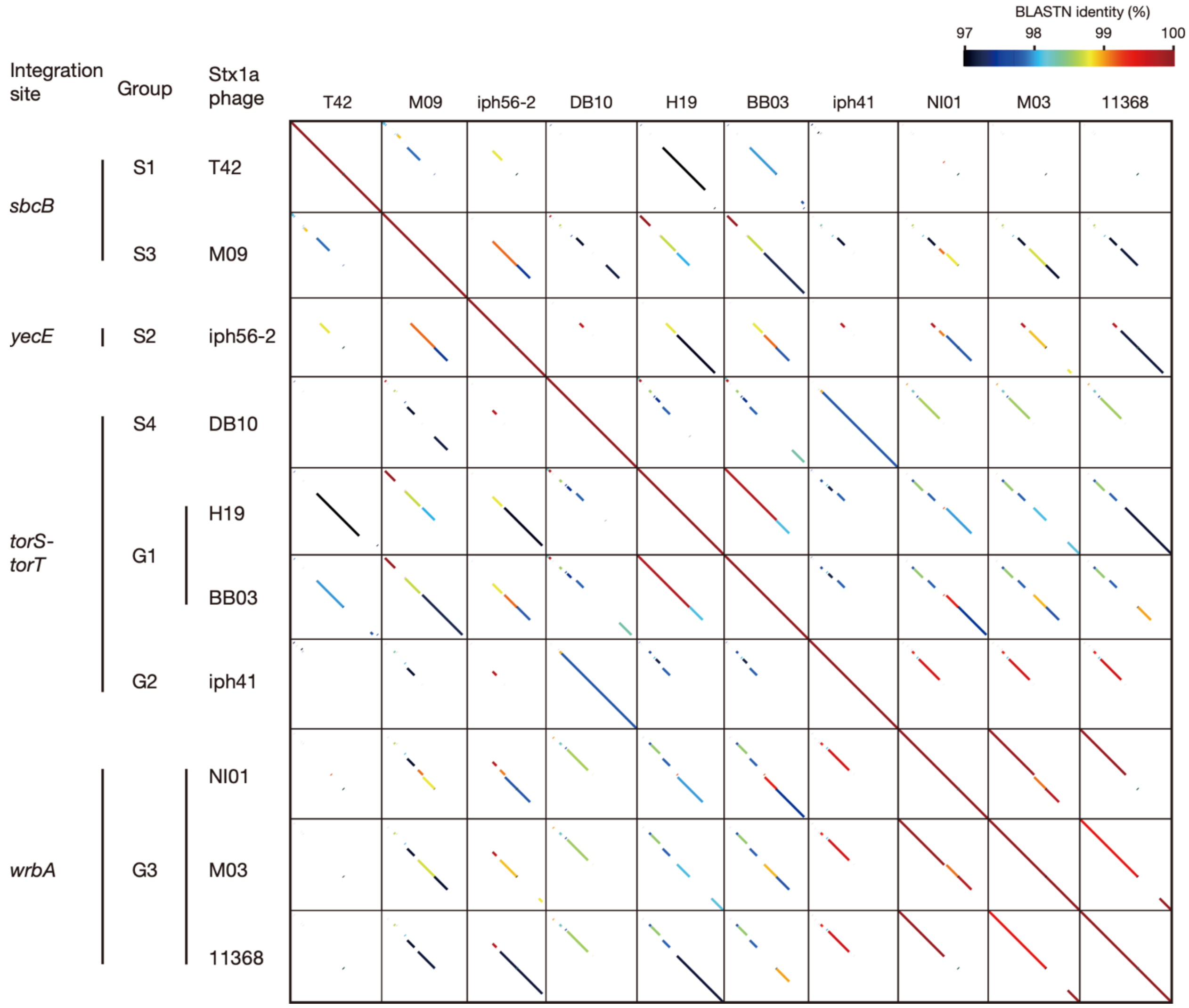

Figure S3. Sequence variation between representative Stx1a phages in O26:H11 ST21 strains.
Dot plot presentation of the sequence similarity between 10 Stx1a phages (six phages representing each of three phage groups [two G1 phages, one G2 phage, and three G3 phages] and four singleton phages) is shown with a heatmap (>97% sequence identity).

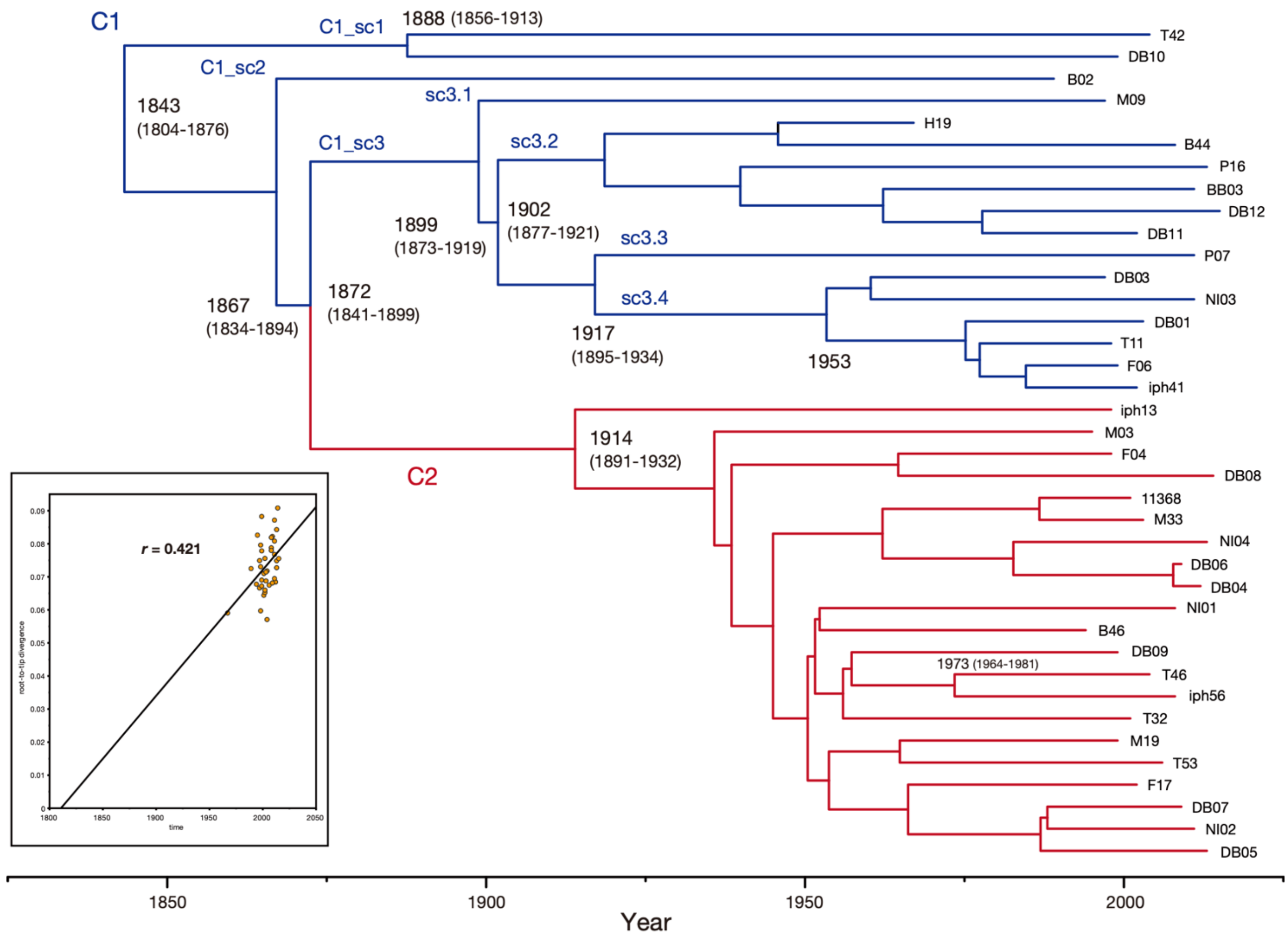

Figure S4. A maximum clade credibility tree of O26:H11 ST21 strains inferred by Bayesian evolutionary analysis.

The result of regression analysis of the root-to-tip distance against the sampling date shown in the inset indicates a positive correlation between genetic distance and sampling date. The time-calibrated phylogenetic tree was reconstructed using BEAST based on the concatenated 3,311 recombination-free SNPs. The time to the most recent common ancestor (TMRCA) of each ST21 clade or subclade is shown in the tree with 95% highest posterior density (HPD) indicated in parentheses. Note that the topology of the tree was identical to that of the ML tree shown in Fig. 1 in the main text.

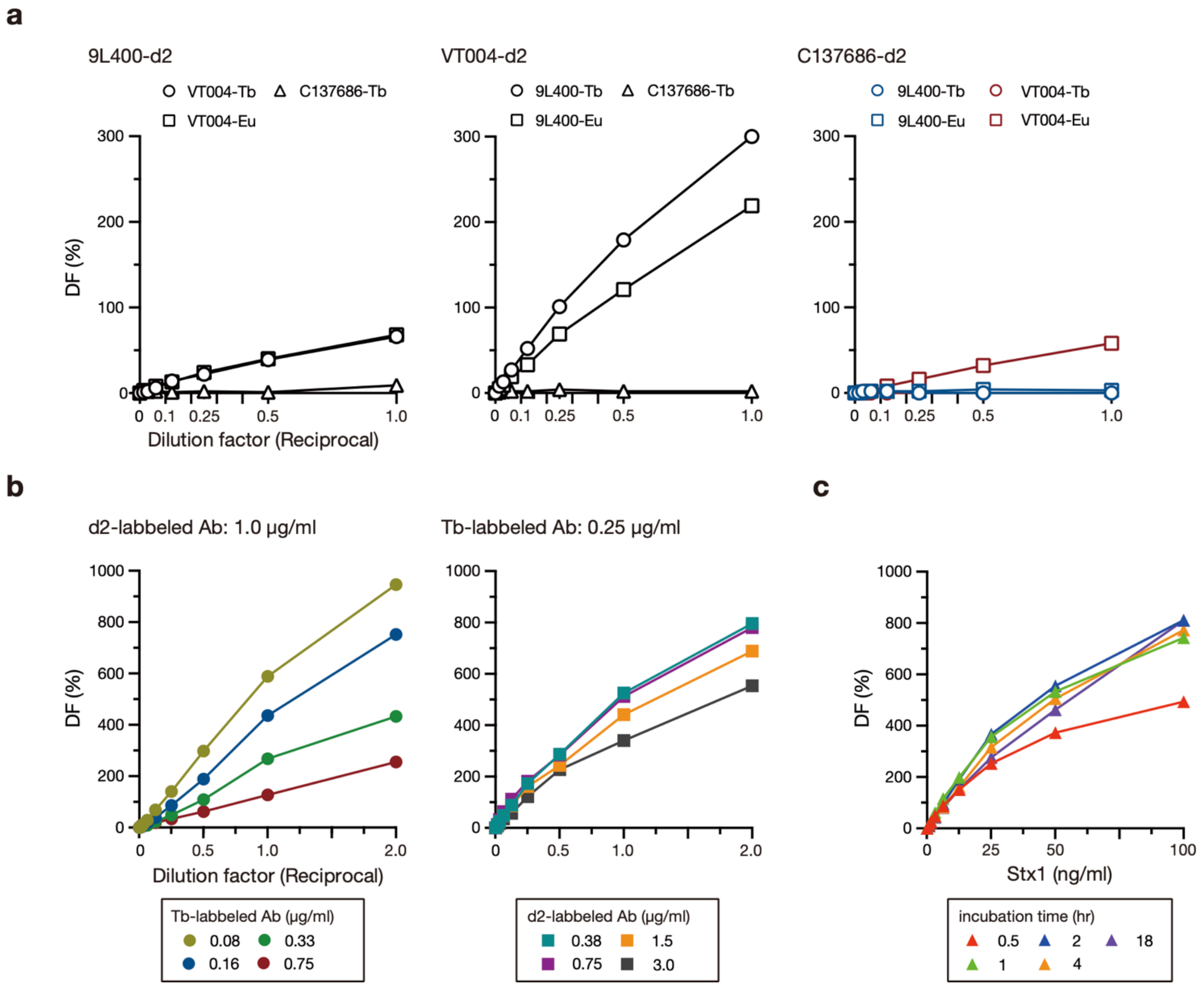

Figure S5. Development and optimization of the homogeneous time-resolved fluorescence resonance energy transfer (HTRF) assay for quantification of Stx1.

The HTRF assay conditions were optimized by selecting the most suitable monoclonal antibody (mAb) pair (a) and determining the most appropriate mAb concentrations (b) and incubation time (c). The concentration of Stx1 (commercially available lyophilized Stx1; see Methods) used in each assay is presented as the reciprocal of the final dilution factor on the x-axis in Panels (a) and (b). Delta F (DF) values were used as the signal intensities of each reaction. Incubation times of the assays shown in Panels (a) and (b) were 2 hr. All data are presented as the mean values (a, b: n=3; c: n=5).

a

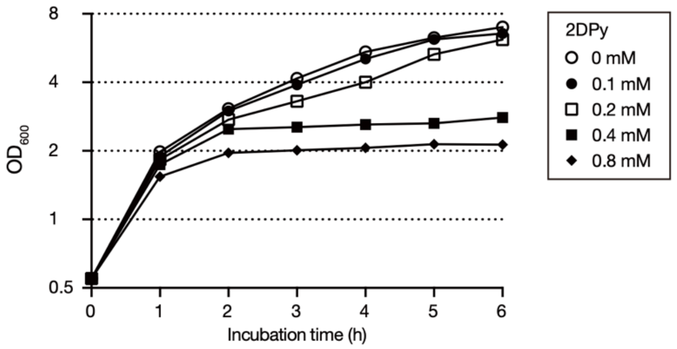

2DPy (mM)	Stx1 production (ng/ml/OD_{600})
0	62
0.1	520
0.2	870
0.4	500
0.8	660

b

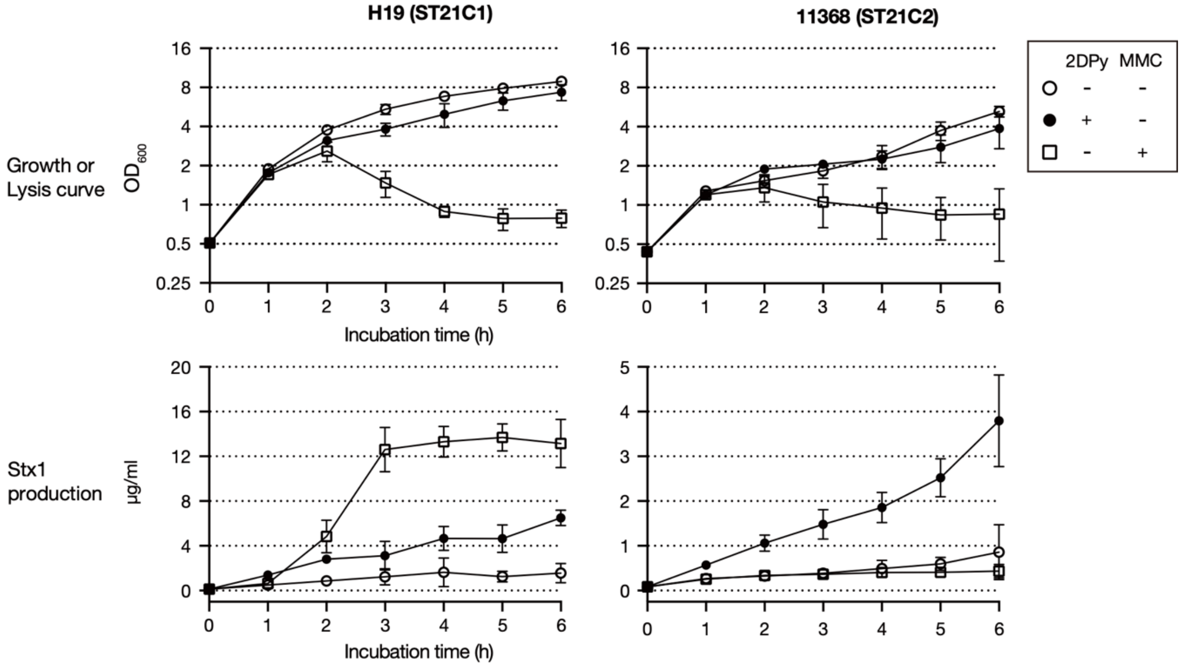

Figure S6. Optimization of assay conditions for Stx1 production.

(a) Growth curves of strain H19 in MS broth containing various concentrations of 2,2'-dipyridyl (2DPy). Bacterial cells were grown to mid-log phase at 37 °C with shaking. After adding 2DPy to the culture at a final concentration of 0, 0.1, 0.2, 0.4, or 0.8 mM, the OD_{600} of each culture was measured every hour for 6 hr. The OD_{600} is displayed on a logarithmic scale. In the right table, the Stx1 concentrations in each culture after 6 hr of incubation are shown as a relative value to OD_{600} (n=1). Based on the results of this analysis, we decided to employ 0.2 mM 2DPy in the Stx1 production assay. This concentration is the same as that previously described [Calderwood and Mekalanos, *J Bacteriol*, 169(10), 4759-4764 (1987)]. (b) Growth and lysis curves and the temporal changes in Stx1 concentrations are shown for each culture of two strains, H19 (ST21C1) and 11368 (ST21C2). Both strains were grown in MS broth or MS broth containing 0.2 mM 2DPy or 0.5 µg/ml MMC. The data of Stx1 production are the same as that shown in Fig. 4, except that the data under the presence of both 2DPy- and MMC are not included in this figure. All values are shown as the mean values with standard deviations of biological triplicates. In both strains, nearly maximum cell lysis and the highest Stx1 production level were observed 4 hr after adding MMC. Therefore, we decided to prepare the samples for the Stx1 production assay at 4 hr for both 2DPy and MMC treatments.

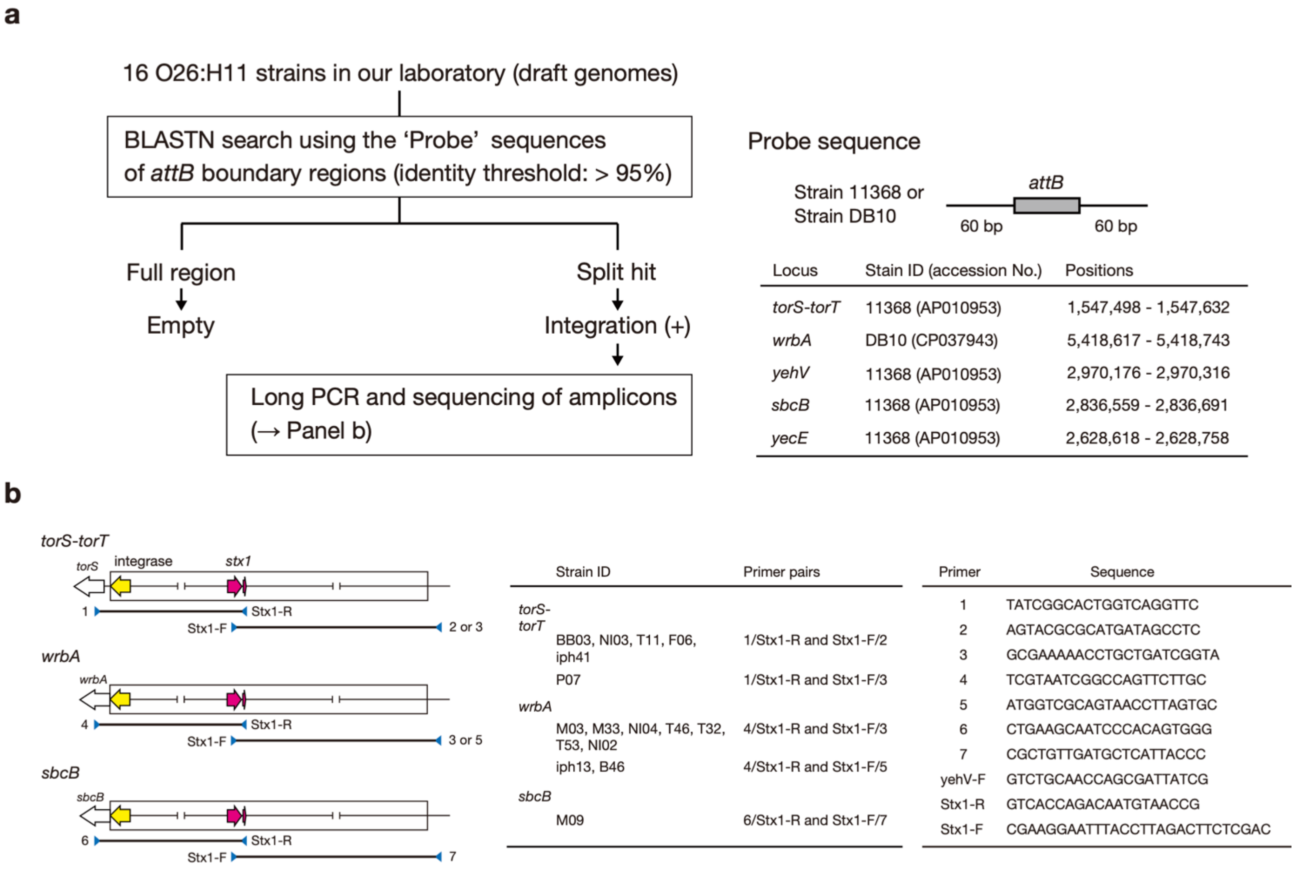

Locus	Stain ID (accession No.)	Positions
torS-torT	11368 (AP010953)	1,547,498 - 1,547,632
wrbA	DB10 (CP037943)	5,418,617 - 5,418,743
yehV	11368 (AP010953)	2,970,176 - 2,970,316
sbcB	11368 (AP010953)	2,836,559 - 2,836,691
yecE	11368 (AP010953)	2,628,618 - 2,628,758

	Strain ID	Primer pairs
torS-torT		
	BB03, NI03, T11, F06, iph41	1/Stx1-R and Stx1-F/2
	P07	1/Stx1-R and Stx1-F/3
wrbA		
	M03, M33, NI04, T46, T32, T53, NI02	4/Stx1-R and Stx1-F/3
	iph13, B46	4/Stx1-R and Stx1-F/5
sbcB		
	M09	6/Stx1-R and Stx1-F/7

Primer	Sequence
1	TATCGGCACTGGTCAGGTTC
2	AGTACGCGCATGATAGCCTC
3	GCGAAAAACCTGCTGATCGGTA
4	TCGTAATCGGCCAGTTCTTGC
5	ATGGTCGCAGTAACCTTAGTGC
6	CTGAAGCAATCCCACAGTGGG
7	CGCTGTTGATGCTCATTACCC
yehV-F	GTCTGCAACCAGCGATTATCG
Stx1-R	GTCACCAGACAATGTAACCG
Stx1-F	CGAAGGAATTTACCTTAGACTTCTCGAC

Figure S7. Procedures to determine the integration of Stx1a phages into the *torS-torT*, *wrbA*, *sbcB*, or *yecE* locus in draft genomes and the entire genome sequences of the Stx1a phages.

(a) Determination of phage integration sites. Draft genomes of 16 O26:H11 strains were searched by BLASTN (>95% identity threshold) using the *attB*-containing sequences of the known Stx phage integration sites (*torS-torT*, *wrbA*, *yehV*, *sbcB*, and *yecE*) as queries. Each query sequence ('probe' sequences) was composed of the *attB* sequence (15 bp at *torS-torT*, 7 bp at *wrbA*, 21 bp at *yehV*, 13 bp at *sbcB*, and 21 bp at *yecE*) and their sequences (60 bp each). Phage integration at each locus was considered positive when the probe sequence was found to be split by some integration. Phage integrations in all but one site were detected by this procedure. In strain NI03, the split probe sequences for the *wrbA* locus were detected in the same scaffold. Based on the annotation of this scaffold, we confirmed the integration of a non-Stx phage into the *wrbA* locus of this strain. (b) Long PCR analysis and sequence determination of Stx1a phage genomes. When the presence of Stx1 phage was confirmed by long PCR, the Stx1a prophage region was amplified by two long PCRs as indicated. By sequencing the PCR products using Illumina MiSeq, the sequence of the entire prophage region was determined. Phage integration into *yehV* was detected in seven strains, but no amplicons were obtained by PCR using yehV-F and Stx1-R primers.
